# Inflammation-Modulating Biomedical Interventions for Diabetic Wound Healing: An Overview of Preclinical and Clinical Studies

**DOI:** 10.1021/acsomega.4c02251

**Published:** 2024-11-01

**Authors:** Nouf N. Mahmoud, Salma Hamad, Sawsan Shraim

**Affiliations:** † Faculty of Pharmacy, Al-Zaytoonah University of Jordan, Amman 11733, Jordan; ‡ Department of Biomedical Sciences, College of Health Sciences, QU Health, Qatar University, Doha 2713, Qatar; § International School of London Qatar, Doha 18511, Qatar

## Abstract

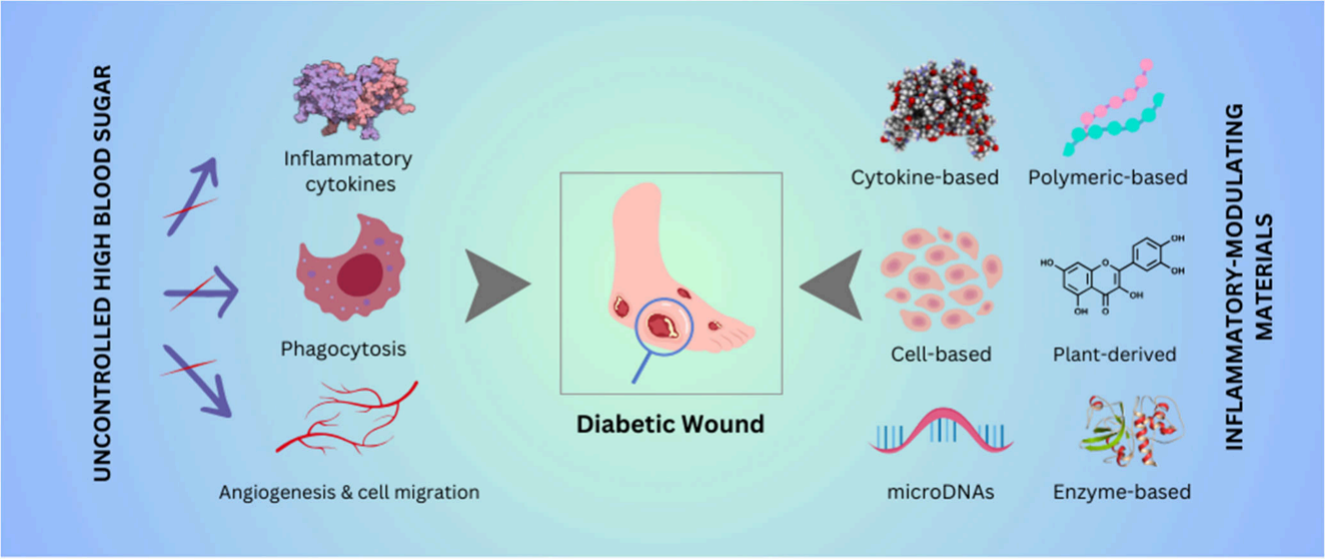

A diabetic wound exemplifies the challenge of chronic, nonhealing wounds. Elevated blood sugar levels in diabetes profoundly disrupt macrophage function, impairing crucial activities such as phagocytosis, immune response, cell migration, and blood vessel formation, all essential for effective wound healing. Moreover, the persistent presence of pro-inflammatory cytokines and reactive oxygen species, coupled with a decrease in anti-inflammatory factors, exacerbates the delay in wound healing associated with diabetes. This review emphasizes the dysfunctional inflammatory responses underlying diabetic wounds and explores preclinical studies of inflammation-modulating bioactives and biomaterials that show promise in expediting diabetic wound healing. Additionally, this review provides an overview of selected clinical studies employing biomaterials and bioactive molecules, shedding light on the gap between extensive preclinical research and limited clinical studies in this field.

## 1 Introduction

Diabetic wounds represent a significant and increasingly prevalent complication among individuals with diabetes mellitus, affecting millions worldwide annually. According to the International Diabetes Federation’s report in 2021, the global population of adults diagnosed with diabetes stood at 537 million.^1^ Among this population, approximately 19% to 34% are expected to develop diabetic wounds at some point in their lives.^2,3^

The complicated pathophysiology underlying diabetic wound healing involves the formation of advanced glycation end-products and a dysregulated inflammatory response.^4^ In particular, the dysfunctional interplay between classical pro-inflammatory macrophages (M1) and resolving macrophages (M2) influences the healing of diabetic wounds. Elevated glucose levels in diabetes disrupt macrophage polarization, perpetuating a state of chronic inflammation and impeding the transition to the reparative phase. This aberrant immune response contributes to delayed wound closure, increased susceptibility to infections, and heightened risk of amputations in affected individuals.^4^

Prolonged and persistent exposure to elevated glucose levels is linked to alterations in cellular functions, proliferation, differentiation and morphology of keratinocytes, shedding light on an additional mechanism through which hyperglycemia can impact wound healing in diabetes.^5^ Furthermore, compromised immune function, such as reduced chemotaxis, phagocytosis, and bacterial killing, significantly contribute to impaired wound healing as a complication of uncontrolled diabetes.^6,7^ Successfully addressing the underlying molecular pathways that contribute to diabetic wounds necessitates developing innovative approaches that target inflammation at its core. Recent advancements in bioactive, biomaterials and cytokine-based therapies offer promising therapies/interventions for modulating the inflammatory environment of diabetic wounds, promoting enhanced healing outcomes. By leveraging bioinspired materials, such as hydrogels incorporating natural polysaccharides or protein-based scaffolds and others, researchers aim to create microenvironments conducive to macrophage polarization toward the pro-reparative M2 phenotype.^8^ Furthermore, cytokine-based interventions targeting key inflammatory mediators hold the potential to orchestrate a favorable immune response, facilitate tissue regeneration, and expedite wound closure in diabetic individuals.^9^

This review explores the multifaceted contributions of macrophages in diabetic wound healing, elucidates the dysregulated inflammatory pathways characteristic of diabetic wounds, and investigates the therapeutic potential of bioactive and biomaterials in modulating inflammation to enhance diabetic wound healing across preclinical and clinical studies.

## 2 Overview of Wound Healing and Unhealed Diabetic Wounds

The process of wound healing is a sophisticated interaction involving multiple stages, characterized by communication among various cell types such as fibroblasts, keratinocytes, and immune cells. Molecular regulators, including cytokines, chemokines, and growth factors, govern this intricate coordination.^10^ It begins with hemostasis, which aims to stop bleeding and prevent microbial intrusion into the injured area. Subsequently, this stage transitions into an inflammatory phase, where the injury intensifies the body’s inflammatory response, causing the recruitment of immune cells. Initially, neutrophils and macrophages are prominent among these pro-inflammatory cells, tasked with removing debris and pathogens while releasing growth factors, cytokines, and other signaling molecules.^11−13^ Key pro-inflammatory cytokines, such as TNF-α, IL-1β and IL-6, are pivotal in attracting inflammatory cells to the injury site.^14,15^ The proliferative phase coincides with the inflammatory phase, facilitating the formation of new tissue and blood vessels through angiogenesis and constructing a matrix to fill the wound area. Within the wound site, various growth factors, released by inflammatory cells, draw proliferating fibroblasts to the affected region and promote epithelialization.^16,17^ Fibroblasts, keratinocytes, and macrophages also contribute to angiogenesis by producing angiogenesis and growth factors, which activate endothelial cells.^11^ The remodeling phase enhances extracellular matrix production and strength while diminishing the blood supply to the damaged area.^18,19^ Maintaining a delicate balance between releasing inflammatory, anti-inflammatory, and transition markers is essential for effectively healing and preventing chronic wounds.^20^

Chronic wounds result from delayed healing, requiring more than 12 weeks for healing.^21^ The unhealed wound is associated with several complications, such as functional limitations and infections such as cellulitis, abscess, gangrene, and amputation.^22^

Chronic wounds usually results from impaired hemostasis, inflammation, proliferation, and remodeling phases, and they negatively impact quality of life, morbidity, and mortality.^22^ Chronic wounds are characterized by sustained high levels of pro-inflammatory macrophages, neutrophils, and proteases, which are directly associated with the severity of the wound,^21,23,24^. Excessive inflammation primarily contributes to wound pathology, sustaining chronicity by continuously damaging the wound tissue. Furthermore, persistent high blood glucose and oxidative stress promote dysregulation of inflammatory cells T, B cells, and macrophages, ultimately inhibiting the immunity of diabetic hosts. This dysfunction is followed by extended release of pro-inflammatory cytokines and compromised cellular and vascular reactions.^25−27^ Elevated levels of pro-inflammatory cytokines exacerbate inflammation and promote insulin resistance, leading to a prolonged healing process for chronic wounds.^9^

The pathophysiology of diabetes significantly interferes with one or more phases of the healing process. The combination of vascular pathology and endothelial cell dysfunction in diabetes burdens distal arteries, thereby reducing the distribution of nutrients and oxygen to the wound site. Consequently, this disruption results in abnormal blood flow to the wound, impairing the normal healing process.^28,29^

Moreover, diabetes-related neuropathy, involving impairment in both motor and autonomic fibers, results in an inability to sense pressure or heat. This lack of sensation, combined with persistent hypoxia, further compromises wound healing and creates an optimal environment for chronic infections.^30,31^ These infections often lead to severe complications and increased morbidity, including gangrene and the necessity for amputation.^32,33^

Furthermore, the healing process encounters additional hurdles in diabetic patients, including hemoglobin glycation-induced hypoxia, metabolic deficiencies, and vascular and red blood cell membrane alteration. Hypoxia diminishes the oxygen availability to wounds, while hemoglobin glycation results in inadequate nutrient and oxygen supply, triggering a stress response and further impeding healing.^34−36^

Growth factors are crucial for the different stages of wound healing, including forming the extracellular matrix (ECM). Any disruption, such as reducing growth factor receptors, delays wound healing among diabetic individuals. A substantial decrease in factors involved in ECM formation, including VEGF, TGF-β, PDGF, EGF, FGF, IGF-1, IL-6, and TNF-α, has been observed in diabetic wounds, contributing to the delayed healing process,^22^. These factors promote vascularization, angiogenesis, and ECM formation while impeding ECM degradation. TGF-β is crucial in recruiting and stimulating inflammatory cells and producing growth factors, and a diminished concentration of TGF-β has been observed in diabetic wounds. Additionally, matrix metalloproteinases (MMPs) are essential for wound debridement and the stages of angiogenesis, epithelialization, and ECM remodeling. Studies indicate that elevated glucose levels affect MMP levels and expression by forming glycation products due to the sustained presence of pro-inflammatory cytokines. Consequently, this disrupts vital growth factors and ECM proteins necessary for effective wound healing.^37^ Other factors related to collagen production, deposition, and epidermal nerves delay the healing of diabetic wounds.^38^

## 3 Influence of Inflammation Dysfunction on Diabetic Wound Healing

Macrophages play a vital role and are a center of interest in diabetic wound healing. Classical pro-inflammatory M1 macrophages are deemed pro-inflammatory due to their response to pro-inflammatory cytokines like TNF and lipopolysaccharide. To eradicate pathogens and cellular debris, these cells produce pro-inflammatory cytokines like IL-6, IL-1β, TNF-α, CXCL9, CXCL10, ROS, and nitric oxide. Concurrently, they promote the proliferation of fibroblasts and keratinocytes, thereby orchestrating the subsequent phases of the repair process. M2 macrophages, the nonclassical macrophages activated by IL-10 and IL-4 and anti-inflammatory cytokines, promote the TGF-β and IGF growth factors release.^25,39,40^

In typical wound healing, M1 macrophages initially dominate during the early stages of the wound, followed by a shift toward M2 macrophages. However, through various mechanisms, heightened glucose levels in the bloodstream have been found to impede the polarization of macrophages. When exposed to elevated glucose levels, M1 macrophages decrease the expression of matrix metalloproteinases 1 (MMP1) and increase the release of several pro-inflammatory cytokines, such as IL-6, IL-1 and TNF-α, while showing relatively low concentrations of anti-inflammatory cytokines like IL-10, TGF-β, and IGF-1.^9^ These alterations lead to prolonged inflammation and a heightened presence of M1 macrophages, which hinder the migration of keratinocytes, fibroblasts, and endothelial cells, resulting in delayed wound healing, sustained M1 macrophage polarization, and chronic inflammation.^41−43^

The mechanisms underlying the modulation of macrophage polarization under hyperglycemic conditions have been elucidated in various studies. Notably, the NF-κB pathway plays a significant role. Chronic hyperglycemia has been shown to enhance NF-κB pathway activation, leading to accelerated production of advanced glycation end products (AGEs). Consequently, this results in cell damage and elevated local levels of IL-1β and TNF-α. The accumulation of AGEs induces intracellular reactive oxygen species (ROS) production, and activates the gene expression of pro-inflammatory nuclear transcription factor NF-κB, thereby triggering pro-inflammatory signaling and promoting macrophage M1 polarization.^44^ Moreover, chronic hyperglycemia has sensitized macrophages to cytokine stimulation, contributing to dysregulated macrophage responses. Therefore, they release higher levels of pro-inflammatory cytokines and extend the inflammatory phase within the wound environment.^45^ Additionally, high glucose levels have disrupted bactericidal and phagocytic activities of the macrophages by reducing their glycolytic capacity and reserve.^46,47^

In diabetic wounds, elevated levels of pro-inflammatory cytokines amplify the persistent production of inflammatory cytokines, proteases, and ROS, causing ECM degradation.^40,48^

An increased quantity of macrophages leads to a higher proportion of these cells becoming classically activated. This activation, in turn, attracts more macrophages, creating a repetitive cycle. As a result, an infected wound experience an overactivation of M1 macrophages. This disruption in the phagocytic process significantly impedes the healing process and elevates the risk of infection.^49^ Individuals with diabetes face a heightened susceptibility to infections, and the risk of hospitalization of individuals with foot ulcer infections increases by 50-fold. About 5% and 20–30% of patients with a diabetic foot ulcer infection necessitate a major amputation or minor amputation, respectively. The presence of a wide variety of resistant bacteria, a significant microbial burden, and biofilms leads to the increased expression of host genes linked to inflammation in the wound tissue.^50,51^

Moreover, the hindered shift from M1 to M2 macrophages correlates with diminished angiogenesis, reduced collagen deposition, diminished phagocytic activity, compromised defense against infection, decreased nitric oxide production, and an increased secretion of pro-inflammatory cytokines, and consequently, significantly inhibited wound healing.^40,52,53^

Elevated glucose levels and constricted blood vessels lead to diminished blood circulation at the wound site, which has an impact on immune cell function and prolongs inflammatory phases. This scenario contributes to delayed healing and development of chronic wounds prone to infections.^54,55^

Research indicates that fibroblasts and vascular endothelial cells exhibited increased ROS levels, lipid peroxidation products, and proteins associated with ferroptosis when exposed to high glucose concentrations in vitro. Additionally, they demonstrated decreased survival and impaired migration under these conditions. Notably, the adverse effects of high glucose were significantly mitigated when treated with the ferroptosis inhibitor.^56^

**Figure 1:**
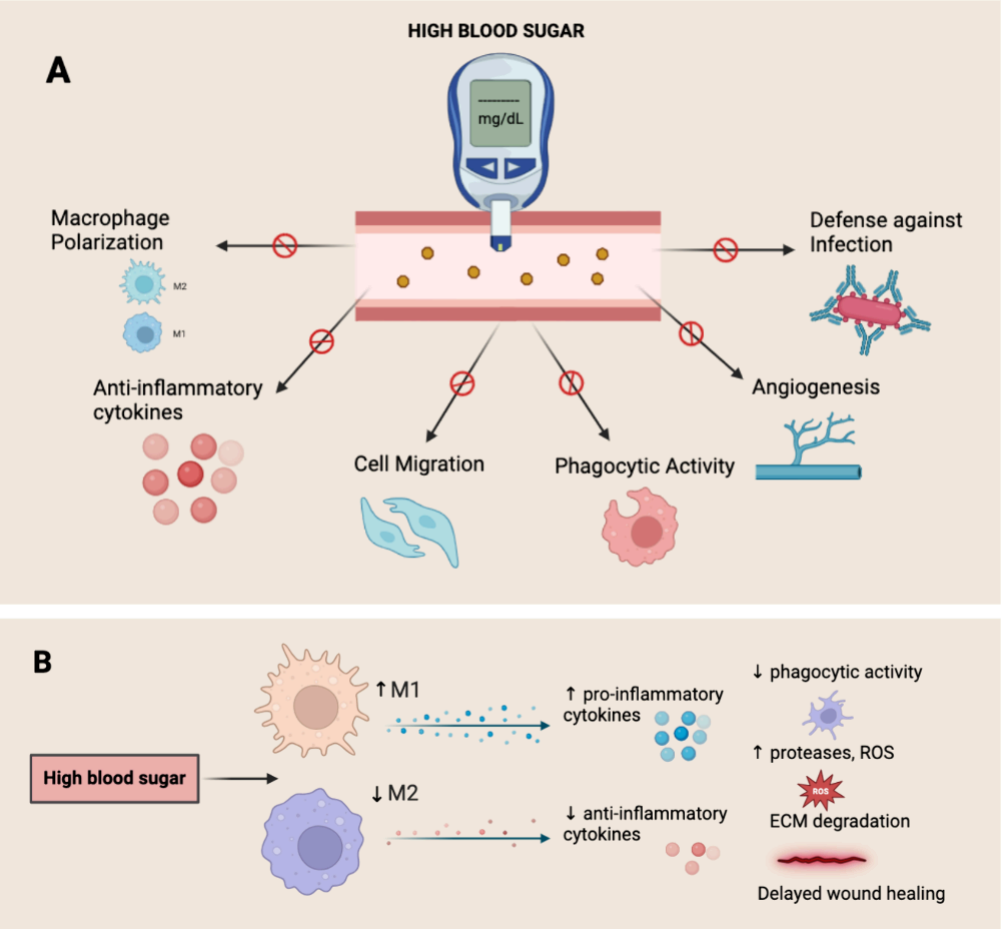
(A) An illustration depicts how persistent high blood sugar levels diminish crucial activities necessary for effective wound healing and contribute to the formation of chronic wounds. (B) An illustration depicts the consequences of the hindered transition from M1 to M2 macrophages as a result of diabetes, thereby delaying the healing process of diabetic wounds. Created with BioRender.com.

Figure 1A illustrates how high blood sugar contributes to the formation of unhealed chronic wounds, while Figure 1B demonstrates the consequences of the hindered transition of macrophages from M1 to M2, resulting in the unhealing of diabetic wounds.

## 4 Bioactive/Bioinspired Materials for Modulating Inflammation in Diabetic Wound Healing-Preclinical Studies

As per the latest data from the National Library of Medicine, the global annual occurrence of diabetic wounds ranges from 9.1 to 26.1 million. About 15 to 25% of individuals diagnosed with diabetes mellitus will encounter a diabetic foot ulcer at some point in their lives.^57^ Given the continual increase in newly diagnosed diabetes cases each year, there is an anticipation of a corresponding rise in the prevalence of diabetic foot ulcers.

Due to the chronic and intricate nature of the pathophysiology associated with diabetic wounds, there is a growing imperative to devise innovative technologies and materials that can effectively address this highly complex process. Beyond traditional treatments that target macro factors and symptoms like pressure and infections, there is an escalating focus on addressing the molecular facets of diabetic wounds. Specifically, interventions directed at sustained inflammation on the molecular level have attracted significant attention. This approach targets specific cellular responses and enhances immunity, providing a more efficacious resolution of persistent inflammation.^58^

Bioactive/bioinspired materials play a pivotal role in aiding the healing of diabetic wounds by mitigating several mechanisms, including inflammation. Below are several examples of the most prevalent and extensively researched bioactive/biomaterials for treating diabetic wounds:

### 4.1 Cytokine and Chemokine-Based Therapies

Various pro- and anti-inflammatory cytokines interact and coordinate the wound healing process. As discussed earlier, diabetes disrupts this balance, among other mechanisms, causing a significant accumulation of pro-inflammatory cytokines at the expense of anti-inflammatory ones. Numerous therapeutic approaches aim to counteract this imbalance by either producing antibodies against pro-inflammatory cytokines or promoting the production/release/concentration of the anti-inflammatory ones. Below are several examples of the most prevalent and extensively researched cytokines and chemokines-based therapies for treating diabetic wounds:

#### IL-22

Several immune cell varieties produce interleukin-22 (IL-22), which is critical in early wound healing. Injury-induced responses prompt IL-22 expression, stimulating cell proliferation, fostering cell migration, and promoting pro-inflammatory gene expression, ultimately triggering tissue regenerations.^59,60^ In a previous study, treating wounds with IL-22 has been shown to stimulate keratinocytes and fibroblast proliferation, contributing to improved wound contraction and enhanced collagen production.^61^ The administration of IL-22 has notably enhanced the healing of diabetic wounds; this acceleration is credited to the facilitation of re-epithelialization and the stimulation of keratinocyte proliferation. Furthermore, the study has shown that IL-22 can directly prompt keratinocyte migration and proliferation.^62^ IL-22 has modulated the keratinocyte differentiation and VEGF production as a mechanism of the observed epidermal hyperplasia in a human epidermis granulation tissue and vascularization.^63^

A favorable impact of IL-22 was observed in studies utilizing cultured human colon adenocarcinoma cells or cultured keratinocytes by enhancing cell proliferation or migration.^64,65^ Other studies further support the positive contribution of IL-22-based therapies in diabetic wound healing.^62,66^

#### IL-1β

By stimulating inflammation, IL-1β increases the mobilization of leukocytes from the bone marrow and prompts the liver to secrete acute-phase proteins. Consequently, neutralizing antibodies or receptor antagonists to inhibit IL-1β function in diabetic wounds may enhance healing.^67^ The Interleukin-1 receptor antagonist (IL-1Ra) effectively regulates IL-1 and thus influences various inflammatory processes. Wounds treated with IL-1Ra exhibited accelerated wound healing, evidenced by reduced neutrophil and macrophage infiltration in skin samples taken 21 days postwound in diabetic mice.^68^ Other studies demonstrate the efficacy of IL-1Ra in promoting diabetic wound healing by creating a conducive healing environment through suppression of early inflammatory response and pro-inflammatory mediators and support of anti-inflammatory factors.^42,68−71^ In normal mice, the protein level of pro-inflammatory cytokines (TNF-α and IL-6) has been significantly decreased in wound tissue upon application of IL-1Ra within 48 h after injury and treatment.^72^ Similarly, administering IL-1β antibodies to db/db mice and IL-1R knockout mice has shortened the M1 phase and decreased the expression of TNF-α and IL-6, enhancing the healing process.^73^

#### IL-10

IL-10, an anti-inflammatory cytokine, exerts anti-inflammatory effects and promotes tissue repair by inhibiting the production of pro-inflammatory cytokines such as TNF-α, IL-6, and IL-1β, suppressing the activity of macrophages and T cells and enhancing the proliferation and migration of fibroblasts, supporting the remodeling phase of wound healing. Nonhealing wounds have low concentrations of IL-10 at their margins, suggesting the crucial role of IL-10 anti-inflammatory cytokine.^74,75^ Various approaches exist to enhance IL-10 expression. For instance, applying curcumin topically on wounds of diabetic rats has been associated with elevated levels of IL-10 production, leading to improved formation of granulation tissue and wound closure.^76^ Another potential method involves utilizing nano micelles of N-acyl LMW chitosan as a gene delivery vector engineered to enhance the expression of IL-10 in diabetic rats, significantly elevated concentration of IL-10, and reduced concentration of some other pro-inflammatory cytokines.^77^

#### IL-33

IL-33 contributes to wound healing by enhancing macrophage polarization and reducing inflammation. Consequently, IL-33 is a promising therapeutic agent for diabetic wounds, where immune modulation is compromised and inflammation is persistent.^78,79^

#### CCL2

CCL2, also known as MCP-1 or C–C motif chemokine ligand 2, is a protein released by epithelial cells, macrophages, and endothelial cells and stimulates angiogenesis, cell proliferation and migration of immune cells to the wound.^80,81^ In the inflammatory and proliferation phases of wound healing, neutrophils release CCL2 and other chemokines, which recruit macrophages to the wound to promote monocyte chemotaxis, migration of more macrophages to the wound, and promotion of angiogenesis.^82^ The role of CCL2 in the remodeling phase was demonstrated in vitro by promoting ECM degradation by increasing the release of MMP-1 and TIMP-1 in vitro.^83^ It has been shown that early stage diabetic wounds display a paradoxical decrease in crucial macrophage response attributed to diminished expression of the CCL2 chemokine.^84^ Crucially, a single administration of the chemoattractant CCL2 has demonstrated considerable effectiveness in enhancing diabetic wound healing by restoring the kinetics of macrophage and promoting transition into the following healing stage without prolonged retention in the inflammatory stage.^84^

Interventions targeting CCL2 have been shown to enhance wound healing in diabetic conditions. For instance, Wood et al. demonstrated that postinjury treatment with CCL2 significantly enhanced macrophage infiltration and enhanced recovery of diabetic wounds.^84^ Likewise, significant improvement of various markers of wound healing in diabetic wounds has been shown following the application of CCL2, such as wound closure, collagen accumulation, increased expression of vascular endothelial growth factor (VEGF), and transforming growth factor-β (TGF-β).^85^ Moreover, the administration of CCL2 through local injection into dermal wounds has been observed to improve the engraftment of mesenchymal stem cells during wound healing.^81^ Furthermore, applying CCL2 topically emerges as a viable therapeutic strategy for treating patients with diabetes by facilitating neovascularization and collagen accumulation at skin wound sites.^86^

Nevertheless, translating cytokines into clinically effective and safe wound treatments is challenging due to their short half-life, rapid degradation, unpredictable signaling, and side effects.^78^ Among the various novel delivery systems investigated to improve the effectiveness of cytokines, hydrogels have attracted interest due to their ability to encapsulate and deliver bioactive molecules, protect against infections, and maintain moisture balance.^87^ However, many hydrogels result in incomplete degradation due to synthetic organic polymers. Therefore, DNA hydrogels, composed of polymeric chains, have recently garnered attention for their enhanced biocompatibility and biodegradability.^78^ For example, a recent study demonstrated the development of an IL-33 cytogel by incorporating IL-33 into cross-linked DNA hydrogels. The developed IL-33 hydrogel showed sustained release of IL-33, enhanced biocompatibility, biodegradability, and antioxidant and anti-inflammatory properties against diabetic wounds in mice, evident by the increased accumulation of M2 macrophages at the wound site.^78^

### 4.2 Polymeric-Based Biomaterials

#### Polysaccharides

Polysaccharides sourced from animals and plants are commonly employed in tissue engineering since they imitate the structure of ECM.

Hyaluronic acid-based biomaterials and Hyaluronic acid cross-linked/conjugated with other biomaterials significantly enhanced the healing of diabetic wounds. For example, acid–based hydrogel significantly accelerated wound closure in vitro and in vivo diabetic models by enhancing angiogenesis through the expression of VEGF and modulating the inflammatory response by promoting 1L-10 and reducing IL-6 cytokines.^88^ Various published studies demonstrated that hyaluronic acid cross-linked with multiple biomaterials such as chitosan, alginate, collagen, or curcumin boosted wound closure in diabetic animal models by promoting cell migration, re-epithelization and reducing the release of pro-inflammatory cytokines.^89−92^

Various molecular weights of hyaluronic acid exhibit different biological activities. High-molecular-weight hyaluronic acid is known for its ability to hydrate tissues, stabilize the extracellular matrix structure, and maintain osmotic balance while also possessing anti-inflammatory properties. Conversely, low-molecular-weight hyaluronic acid demonstrates angiogenic potential but can also enhance pro-inflammatory responses.^93^ Consequently, high-molecular-weight hyaluronic acid has been employed in numerous scaffolds for diabetic wound healing. For example, a hydrogel incorporating high-molecular-weight hyaluronic acid and paeoniflorin has significantly promoted cutaneous healing by reducing inflammation, enhancing angiogenesis and re-epithelialization, and facilitating collagen deposition in diabetic mice.^94^ Additionally, a dressing composed of higher molecular weight hyaluronic acid combined with povidone-iodine complex notably accelerated diabetic wound healing by promoting angiogenesis and tissue regeneration while dampening pro-inflammatory reactions.^95^

Glycosaminoglycans, found within the ECM of mammals, support the ECM through cellular hydration and contribute significantly to cell signaling. Furthermore, they profoundly influence chemokine signaling, impacting cellular processes such as proliferation, cell differentiation and migration.^96^ Among glycosaminoglycans, heparin sulfate has effectively reduced levels of chemokines such as CCL1, 2, and 8 in wounds at 5 and 10 postwound in diabetic mice and bacterial-infected diabetic mice, facilitating the transition to the proliferative stage.^97^ Furthermore, a biocompatible methacrylate gelatin hydrogel with snail glycosaminoglycan incorporation showcased remarkable anti-inflammatory and tissue adhesion properties.^98^ Applying this biodegradable hydrogel has significantly accelerated chronic wound healing in a diabetic mouse model.^98^ Other studies revealed that the snail glycosaminoglycan hydrogel effectively reduces inflammation by inhibiting the NF-κB signaling pathway and promoting macrophage polarization toward the M2 phenotype, suppressing the prolonged inflammatory phase.^98^

Brown alga, a seaweed rich in polysaccharides, presents abundant marine resources and a typical example of ECM mimic scaffolds.^99^ Brown alga polysaccharide offers anti-inflammatory, immunity modulation and antioxidant properties.^100^ An electrospun fiber containing brown alga- showed promising wound healing in an animal diabetic wound by reducing TNF-α and IL-6 pro-inflammatory levels, promoting angiogenesis, and accelerating wound closure.^101^

Alginate gel has been demonstrated to modulate the inflammatory response in primary macrophages and fibroblasts extracted from diabetic mice by modulating the expression of cytokines and chemokines such as CXCL4, CXCL5, CCL1, 2, 3, 5, 11, and 12.^102^

#### Protein Silk Fibroin (SF)

SF is intricately involved in all stages of wound healing, operating at both physiological and molecular levels.^103,104^ SF facilitates inflammatory cell infiltration, cell proliferation, and tissue remodeling by activating various cellular signaling pathways.^104^ For example, Park et al. indicated that upon SF activation, protein levels of IKKα, IKKβ, p65, and the degradation of IκBα increased, indicating the activation of the NF-κB signaling pathway. Additionally, exposure to SF resulted in elevated expression of VEGF, fibronectin, cyclin D1, and vimentin.^105^ A remarkably viscous nanosilk fibroin solution demonstrated the ability to enhance the tensile strength of human skin upon application and diminish IL-6 levels in diabetic wounds.^106^ Angiogenesis at an early stage of healing in acute and diabetic wounds has been shown upon treatment with nanofibrous matrices composed of various silk compositions.^107^ Moreover, Fibroin has been combined with several materials to foster its physicochemical and biological properties. For instance, the Fibroin’s mechanical, absorption, biological, and biocompatibility have been enhanced upon incorporating glucose as a plasticizer.^108^ Furthermore, β-glucan Paramylon has been combined with Fibroin to enhance the immune response and the wound-healing potential of Fibroin bioactive film.^109^ Besides, the mechanical properties and cellular responses of silk fibroin have been fostered by the incorporation of egg white at different ratios.^110^

### 4.3 Cell-Based Therapies

#### Stem Cells

Stem cells represent a plentiful reservoir of extracellular matrix proteins, growth factors, and cells that have already found application across diverse fields within tissue engineering and regenerative medicine.

Mesenchymal stromal cells (MSCs) have attracted attention due to their pivotal role in wound healing, underscored by their innate self-renewal capacity and multifaceted abilities in immunomodulation, anti-inflammation, antifibrosis, angiogenesis, and therapy.^111^ The primary mechanism underlying MSC-mediated diabetic wound healing involves modulating the secretion of angiogenic growth factors, inflammatory cytokines, and extracellular vesicles. These elements have synergistically enhanced re-epithelialization, promoted granulation tissue formation, and facilitated angiogenesis.^112^ Intradermal administration of MSCs to diabetic wounds has expedited re-epithelialization and granulation tissue formation through modulating the secretion of inflammatory chemokines and cytokines, like IL-1, 6, 8, TNF-α, CCL2, PGE2, and IL-10.^113−115^ Another study demonstrated accelerated wound healing in diabetic mice treated with intradermal injection of amniotic mesenchymal stem cells by enhancing the expression of angiogenic factors such as EGF, IGF-1 and IL-8 and promoting cell differentiation.^116^ They also enhanced angiogenesis, re-epithelialization and increased growth factors such as PDGF-A and HGF levels in diabetic rats.^117^ Likewise, mesenchymal stem cells incorporated in a dermal matrix of collagen/chitosan activated the Wnt signaling pathway, thus promoting cell proliferation and differentiation and accelerating wound healing in diabetic rats.^118^ On the other hand, bone marrow mesenchymal stem cells (BMSC) regulated wound healing by secreting TGF-β and FGF and enhancing cell differentiating like keratinocytes, fibroblasts, and endothelial cells, thereby accelerating angiogenesis, re-epithelialization and formation of granulation tissues.^119,120^ Studies comparing different hydrogels found that human adipose-derived mesenchymal stem cells loaded into PGmatrix exhibited potential anti-inflammatory and promoting early immunosuppressant effects, accelerating diabetic wound healing.^121^

Adipose-derived stem cells (ADSC), with their fibroblastic morphology, enhance granulation, epithelialization, tissue formation and production of growth factors such as TGF-β1, TGF-β3, VEGF, HGF, and FGF2. Additionally, they demonstrate potential in augmenting angiogenesis.^122,123^ Moreover, human amniotic membrane epithelial cells (hAEC) have antioxidant, anti-inflammatory, and anticancer activities, and they have shown accelerated healing in diabetic mice through re-epithelialization and production of growth factors and cytokines such as EGF, PDGF-B, IL-8 and CXCL7.^124^

### 4.4 MicroRNAs (miRNAs)

miRNAs have captured significant attention among the bioactive molecules studied for their potential to promote diabetic wound healing. Intradermal injection miRNA-497 has accelerated wound healing in diabetic mice by reducing pro-inflammatory cytokines such as TNF-α, IL-1β, and IL-6.^125^ Another study explored the impact of miRNA-138 inhibitor on diabetic wounds in rats by modulating the PI3K/AKT and hTERT pathways involved in inflammation modulation, decreasing the level of inflammatory cytokines and increasing the level of anti-inflammatory cytokines.^126^ A recent study demonstrated that miRNA146a-loaded exosomes conjugated to silk fibroin demonstrated anti-inflammatory and regenerative effects and improved diabetic wound healing by reducing inflammation and enhancing tissue repair by targeting the IRAK1 pathway.^127^ In diabetic rat wounds, subcutaneous injection of miRNA-185–5p mimics decreases the expression of CD68, TNF-α, IL-6, p-NF-κB, and ICAM-1 inflammatory mediators, boosting re-epithelization and wound closure.^128^ The administration of miRNA-145a-5p mimics significantly hastened wound healing in diabetic mice by modulating the polarization of macrophages and enhancing the anti-inflammatory activity of M2 macrophages.^129^ Moreover, the efficacy of miRNA-132 in wound healing was demonstrated in diabetic mice by modulating various signaling pathways involved in inflammation, such as the TNF signaling pathway and toll-like receptors.^130^

### 4.5 Enzyme-Based Bioactive

Nanozymes have been found to be extensively used across various biomedical applications, from disease detection to tissue repair and nerve injury treatment.^131^ Their versatility extends to regulating the wound microenvironment, opening new avenues for developing and utilizing wound dressings. Du et al. found that pH-switchable nanozyme cascade catalysis achieves full re-epithelialization of wounds in diabetic mice in 2 weeks, offering promising prospects for clinical diabetic wound treatment.^132^ Enzyme-based materials provide a range of therapeutic possibilities, from antibacterial action to anti-inflammatory effects and wound healing. These materials may utilize single enzyme activities, such as peroxidase or superoxide dismutase, to produce ROS for bacterial biofilm destruction and inflammation reduction, thereby promoting diabetic wound healing.^133−135^ Moreover, enzyme-based materials can expedite tissue repair and regeneration by effectively eliminating reactive oxygen species and reducing inflammatory responses in wounds, facilitating angiogenesis; catalase and glutathione peroxidase are examples of such enzymes.^131^ These materials can also accelerate wound healing by generating essential products for cellular functions, such as nitric oxide, which is crucial in endothelial cell function.^136−138^ Current research focuses on enzyme-based materials with diverse enzymatic activities targeting multiple stages of wound healing. For example, nanocubes of Cu_2_O–Pt exhibit enzyme-like activity, accelerating wound healing in an infected mice model through accelerating cell proliferation, tissue remodeling, and collagen deposition through promoting the VEGF-AKT-ERK1/2 signaling pathway. Furthermore, the nanocubes retarded the expression of IL-1β and TNF-α pro-inflammatory cytokines released by diabetes and bacterial infection.^139^

### 4.6 Plant-Derived-Based Bioactives

#### Curcumin

Curcumin, known for its anti-inflammatory properties, has been shown to inhibit the activity of NF-κB and, consequently, reduce the release of TNF-α and IL-1 pro-inflammatory cytokines. This modulation of NF-κB involves various pathways, including those activated by kinases such as AKT, PI3K, and IKK. NF-κB, long considered sensitive to oxidation, underscores the interplay between oxidation and inflammation in wound healing.^140^ Furthermore, curcumin possesses anti-inflammatory activity by modulating several activities of neutrophils; it has been shown that curcumin promotes phagocytosis, degranulation of neutrophils, enhanced gelatinase activity, and suppresses TLR-4 coreceptors, modulating inflammation.^141,142^

In a previous study, chitosan nanoparticles loaded with curcumin modulated the inflammatory responses through the NF-κB pathway, reduced TNF-a and IL-6 inflammatory markers levels, and enhanced cell migration and angiogenesis in vitro and in vivo, enhancing diabetic wound healing.^143^ Another study demonstrated the wound healing efficacy of nanofibers of curcumin-loaded polycaprolactone-poly(vinyl alcohol)-silk fibroin by showcasing anti-inflammatory and antioxidant activities in vitro and in vivo models.^144^ However, a recent meta-analysis revealed that curcumin contributes to wound healing in diabetic mice/rats by exerting antioxidant activity, enhancing angiogenesis and collagen deposition. Nonetheless, the anti-inflammatory activity remains controversial.^145^

#### Quercetin

The literature emphasizes the role of quercetin in modulating the inflammatory response in diabetic wounds. For instance, studies have demonstrated that quercetin accelerates wound healing in diabetic rats by enhancing the expression of the anti-inflammatory marker IL-10 and growth factors while suppressing the expression of pro-inflammatory cytokines such as TNF-α and IL-1β.^146^ Comparable outcomes have been observed in other studies employing varying concentrations of quercetin.^147^ Abid et al. have demonstrated enhanced wound healing of quercetin-4-formyl phenyl boronic acid complex in diabetic rats by promoting re-epithelization and angiogenesis.^148^

### 4.7 Neuropeptides

#### Substance P (SP)

SP modulates cell migration, proliferation, and vasodilatation.^149^ In diabetic wounds, the level of SP is much lower than in nondiabetic wounds due to increased degradation of SP by an increased level of endopeptidase.^150^ Inhibition of endopeptidase has been found to improve diabetic wound closure, suggesting the positive impact of SP in diabetic wound healing.^151^ Moreover, SP has exhibited the capacity to diminish leukocyte infiltration and impede the enlargement of lymphoids. The administration of SP has been observed to facilitate a shift toward M2 monocytes, which has led to decreased pro-inflammatory cytokines and heightened anti-inflammatory cytokines.^152^ Leal et al. have found that applying SP to diabetic wounds in mice and rabbits resulted in reversing the chronic pro-inflammatory status and promoting the transition of macrophages into M2 phenotype.^153^

### 4.8 Dietary-Based Therapies

#### Camel and Sheep Milk

The healing of diabetic wounds in mice is accelerated by a diet enriched with whey protein derived from camel milk.^154^ Whey proteins contain various components, including lactoferrin (LF), lactalbumin, and other elements, potentially supporting wound healing in diabetic individuals by regulating inflammation, blood glucose levels, oxidative stress, and growth factors.^155,156^ Studies have shown that whey proteins in camel milk regulate the expression of TNF-α and cell death receptor mRNA, facilitating diabetic wound healing.^157^ Furthermore, camel milk whey proteins are effective in wound healing in diabetic mice by modulating pro-inflammatory cytokines and β-defensin levels and mitigating oxidative stress.^158^ Furthermore, camel milk, rich in fatty acids, peptides, LF, and zinc, possesses antibacterial, antioxidant, and antidiabetic properties.^159^ It has been shown that hydroxyproline from camel milk promotes regenerative tissue healing in diabetic mice.^160^ On the other hand, sheep milk, known for its high lactoferrin content, has been shown to alleviate inflammation and protect against microbial infections.^161^ Research by Tang et al. demonstrated that topically applied conjugated linoleic acid in milk fat protects the skin by modulating the inflammatory response.^162^ It has been shown that conjugated linoleic acid significantly inhibited pro-inflammatory cytokines (IL-6, IL-1 β, TNF- α) and the expression of pro-inflammatory enzymes in inflamed skin in mice.^162^ Furthermore, sheep’s milk contains polar lipids, particularly sphingolipids, with bacteriostatic activities that modulate inflammatory responses.^163^

Table 1 summarizes the main bioactive/biomaterials investigated as inflammation-modulating potential therapies for diabetic wounds and their main contributions.

**Table 1:** Summary of the Main Inflammatory-Modulating Bioactive/Biomaterials for Diabetic Wound Healing and Their Contributions

Material	Contribution
Cytokines and Chemokines-Based	
IL-22	- Stimulating cell proliferation and migration.
	- Facilitating re-epithelization and collagen production.
IL-1Ra	- Reducing neutrophil and macrophage infiltration.
	- Modulating the production of inflammatory cytokines.
IL-10	- Suppressing the activity of immunity cells.
	- Inhibiting the production of pro-inflammatory cytokines.
	- Enhancing the proliferation and migration of fibroblast, and supporting tissue remodeling.
IL-33	- Enhancing transition from M1 to M2 transition.
	- Reducing long-term inflammation.
CCL2	- Enhancing production of growth factors.
	- Enhancing macrophage infiltration early and promoting transition into the following healing stages.
	- Facilitating neovascularization and collagen accumulation.
Polymeric-Based	
Polysaccharides (Hyaluronic acid, Glycosaminoglycans, Chitosan, Alginate)	- Modulating the cytokines and chemokines production.
	- Modulating the NF-κB signaling pathway and inflammation.
	- Promoting angiogenesis, and collagen deposition
	- Enhancing the expression of growth factors.
Protein silk fibroin/nanosilk	- Diminishing release of pro-inflammatory cytokines.
	- Facilitating cell proliferation and tissue remodeling.
	- Enhancing angiogenesis
Cell-Based	
Stem cells (MSC, BMSC, ADSC, hAEC)	- Secretion of angiogenic growth factors, immunomodulatory agents, and remodeling molecules.
	- Accelerating re-epithelialization and granulation tissue formation.
	- Augmenting neovascularization and cytokines.
miRNAs and Enzyme-Based	
MicroRNAs mimics	- Modulating signaling inflammatory pathways (e.g., PI3K/AKT)
	- Suppressing expression of pro-inflammatory mediators
Enzyme-based materials	- Eliminating ROS and reducing inflammatory responses.
	- Accelerating angiogenesis, collagen accumulation, cell proliferation and tissue remodeling.
	-Inhibiting the expression of pro-inflammatory cytokines.
Plant-Derived-Based Bioactive	
Curcumin	- Inhibiting the activity of NF-κB, reducing pro-inflammatory cytokines
	- Mitigating inflammation and enhancing angiogenesis.
	- Showcasing antioxidant activity.
Quercetin	- Modulating the expression of inflammatory mediators.
Neuropeptides	
Substance P	-Promoting the transition to M2 monocytes.
	- Modulating levels of inflammatory cytokines.
Dietary-Based Therapies	
Camel/sheep milk constitutes (Whey protein/lactoferrin/fatty acids/polar lipids)	- Regulating inflammation, oxidative stress and growth factors.
	-Showcasing antibacterial and antioxidant activities.
	-Promoting re-epithelization and tissue remodeling.

## 5 Overview of the Clinical Studies Involving Inflammation-Modulating Bioactive/Biomaterials for Diabetic Wounds

The topic of inflammation-modulating bioactive/bioinspired materials for diabetic wound healing is multifaceted and important in medical research and clinical practice. Diabetic wounds present a complex challenge due to the impaired healing processes associated with diabetes, which often lead to chronic wounds, increased risk of infections, and even limb amputations if left untreated. Despite the urgent need for developing effective treatments, the availability of FDA-approved therapies for wound healing, especially for diabetic wounds, is limited. While the number of FDA-approved anticancer drugs is abundant, the treatments available for wound healing are relatively few.^206^ For example, Regranex, a gel containing topically applied human recombinant platelet-derived growth factor-BB, is approved for neuropathic diabetic ulcers. Moreover, only a few biological medical devices have received FDA approval for treating diabetic foot ulcers. These include Dermagraft and Apligraf, both cell-based human skin equivalents or substitutes, and Omnigraft, an animal-derived matrix initially intended for burn treatment. This disparity highlights the significant gap in translating preclinical successes into clinically approved treatments, emphasizing the urgent need for continued research and development in this field.

Basic research and preclinical studies have extensively investigated the potential of cytokines and various biomaterials to mitigate inflammation and promote wound healing in diabetic conditions. Despite promising findings from basic research, translating these discoveries into clinical trials has been limited. The shortage of clinical trials specifically focusing on biomaterials for diabetic wound healing is notable, as evidenced by the relatively low number of studies in biomedical databases like PubMed and Medline. Since 2000, PubMed has published approximately tens of thousands of articles concerning diabetic wounds/ulcers, with clinical trials comprising only a fraction of this body of literature. This discrepancy may stem from various challenges, including the complexity of diabetic wound pathophysiology, heterogeneity in patient populations, and regulatory hurdles associated with clinical trials for medical devices and biomaterials. Furthermore, other challenges in the clinical translation of inflammation-modulating biomaterials for diabetic wound therapy relate to variations in the models used for studying materials, inconsistent analytical methods, and incomplete exploration of wound healing marker profiles commonly utilized to monitor therapeutic efficacy.^164^

Nevertheless, it is worth noting that many of the published clinical trials in biomaterials-based diabetic wound therapy use materials composed of polysaccharides, dermal/tissue substitutes/equivalents, and stem cells.

In clinical investigations concerning polysaccharides, a randomized clinical trial (RCT) was conducted to compare the efficacy of chitosan and nanosilver dressings in treating chronic diabetic wounds in 25 patients. The study revealed a similar percentage reduction of the diabetic-foot-infection score for the chitosan and nanosilver dressing groups (78.1% vs 74.1%, respectively) over 6 weeks with no reported adverse events.^165^ Another double-blinded RCT investigated the additional benefits of combining isosorbide dinitrate spray and chitosan gel for managing diabetic foot ulcers in 68 patients with diabetic foot ulcers. This trial demonstrated the highest percentage of wound closure with the combined treatment (∼ 71%). However, no notable differences were observed between the combination therapy and individual treatment groups concerning immunohistochemical markers.^166^ Moreover, an open randomized clinical study assessed the efficacy of a next-generation chitosan wound dressing composed of chitosan fibers in treating chronic wounds in 90 patients with unhealed chronic wounds, revealing significant reductions in wound area (∼66% vs ∼40%) and lower pain levels in the chitosan group compared to controls (1.1 vs 2.3).^167^ Additionally, an RCT in Iran compared a bioactive dressing containing chitosan to conservative gauze treatment in 85 patients with diabetic foot ulcers. After 21 days, the bioactive dressing group exhibited significantly higher rates of complete wound healing in 29 out of 34 patients and lower infections than controls.^168^ Various clinical studies have demonstrated enhanced wound healing in diabetic wounds upon treatment with other polysaccharide-containing composites/dressings such as hyaluronic acid,^169−172^ alginate,^173,174^ oligosaccharides,^175^ collagen,^176^ cellulose,^177^ and Promogran (a collagen/oxidized regenerated cellulose dressing).^178,179^ For example, in an RCT, 84% of patients with diabetic ulcers showed complete healing upon treatment with a complex of autologous fibroblast and hyaluronic acid compared to 34% of the control group.^171^ Furthermore, prospective RCT patients with diabetic ulcer-treated hyaluronic acid dressing material demonstrated a higher complete healing rate (84.6%), faster healing velocity and shorter time for 50% ulcer size reduction compared to the control group.^172^

Concerning clinical studies involving wound healing mediators/cytokines, an RCT by Lobmann et al. explored the effects of a protease-inhibitory modulating matrix on chronic diabetic foot ulcers in 33 patients. While mRNA levels of matrix metalloproteases (MMPs) and inflammatory cytokines remained unchanged, the treatment group significantly reduced the (MMP)-9/TIMP-2 ratio, enhancing wound healing.^180^ Another study involving 51 patients with chronic diabetic foot ulcers treated over 8 weeks demonstrated that a combination of protease-modulating matrix and growth factors significantly reduced all three ulcer dimensions compared to a single treatment, suggesting enhanced efficacy with combined therapy.^181^

Some studies have shown promising results for tissue substitutes/matrix therapy. For example, an RCT comparing diabetic foot ulcer patients treated with human fibroblast-derived dermal substitute plus standard care to those receiving standard care alone showed a lower incidence of amputation/bone resection in the dermal substitute group compared to the standard care group among 314 patients (8.9% vs 12.6%, respectively).^182^ In a prospective pilot study involving diabetic patients with chronic lower extremity wounds, treatment with graft-jacket tissue matrix, a cultured skin equivalent, resulted in superior rates of complete wound closure, with 12 out of 14 patients achieving closure by week 16, compared to standard wound gel treatment and notable enhancements in wound healing parameters, indicating its promising potential in managing such wounds.^183^ A prospective randomized study assessed the effectiveness of the Graft-jacket tissue matrix in promoting wound closure in diabetic foot ulcers. It showed faster healing with the tissue matrix after one month of treatment, highlighting its potential benefits, especially in cases with poor circulation.^184^ Another RCT revealed that the dermal equivalent significantly stimulated healing and accelerated the epithelization rate in 60 patients with diabetic foot ulcers, suggesting its potential as an effective treatment approach for diabetic foot ulcers.^185^ Furthermore, an RCT indicated that 65.3% of 43 patients with diabetic ulcers demonstrated complete healing upon treatment with autologous dermal and epidermal-based grafts compared to 49.6% of the control group.^186^

The published clinical trials concerning stem cells demonstrated positive, promising results on diabetic wound healing upon utilizing human umbilical cord mesenchymal stem cells (hUC-MSC),^187^ dermal mesenchymal stem cells,^188^ mesenchymal stromal cells,^189^ bone-marrow-derived stem cells (BMMSCs),^190−192^ adipose-derived stem cells (ASC),^193−195^ and bone marrow mononuclear cells (BMMNCs).^196,197^ For example, a pilot clinical study showed that more than 95% of ulcers closed for all patients treated with hUC-MSC within 1.5 months post-treatment. Furthermore, In an RCT of 41 diabetic patients with critical limb ischemia and foot ulcers, BMMSC treatment resulted in faster and more complete ulcer healing than BMMNCs, achieving 100% healing 4 weeks earlier. At 24 weeks, BMMSC transplantation showed superior improvements in limb perfusion parameters, indicating its potential as a more effective therapy for diabetic CLI and foot ulcer management.^190^ An RCT of 59 patients and allogeneic ASC sheets were examined for diabetic foot ulcers. ASC treatment demonstrated superior wound closure rates (73% at week 8, 82% at week 12) compared to controls (47% at week 8, 53% at week 12), with no serious adverse events, indicating their efficacy and safety.^193^

A clinical study of natural-derived compounds (e.g., curcumin and quercetin) demonstrated the efficacy and safety of a nanohydrogel containing quercetin and oleic acid, examined in 56 diabetic patients with lower limb wounds. Results indicated a notable acceleration in wound healing by reducing the wound healing time compared to treatment with hyaluronic acid, suggesting the potential utility of this formulation in wound management.^198^ On the other hand, a clinical trial investigated the impact of nanocurcumin intake on 60 patients with grade 3 diabetic foot ulcers demonstrated that nanocurcumin significantly improved glycemic control, insulin sensitivity, lipid profiles, total antioxidant capacity, and total glutathione levels but did not affect wound healing parameters.^199^

**Figure 2:**
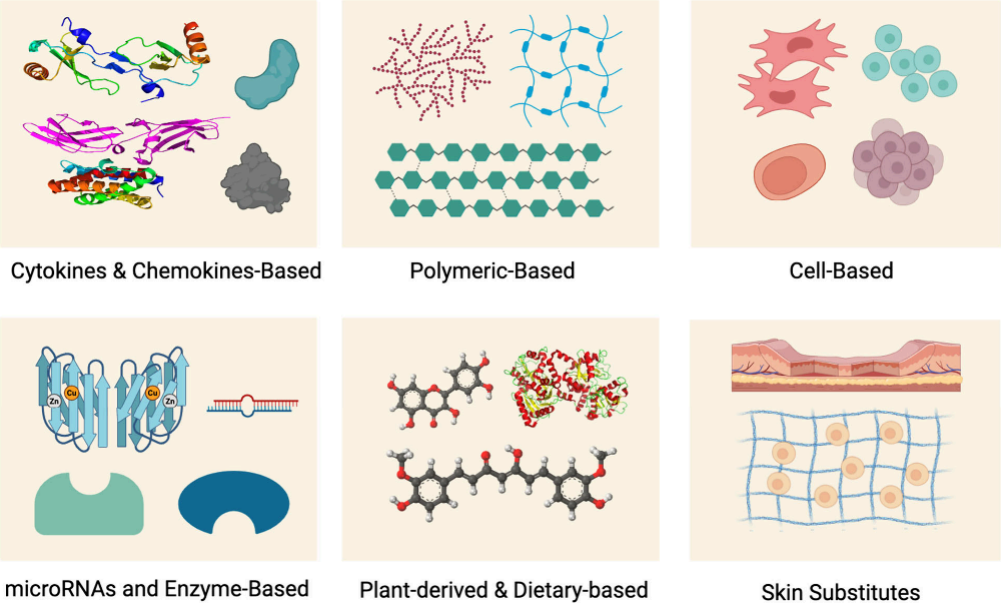
Examples of inflammation-modulating bioactive/bioinspired demonstrated promising wound healing in preclinical and/or clinical studies. Created with BioRender.com.

Figure 2 illustrates examples of bioactive/biomaterials that have promising potential in treating diabetic wound/ulcer in preclinical and clinical studies.

## 6 Recommendations and Future Directions

While significant progress has been made in predicting the role of inflammation-modulating biomaterials in diabetic wound healing, several limitations warrant consideration for future investigations. For example, challenges in current research include the need for more extensive clinical trials to validate the effectiveness and safety of biomaterials for diabetic wound healing. Furthermore, comprehensive research to improve existing potential biomaterials for diabetic wound healing is crucial, particularly in aspects of biocompatibility, mechanical properties, and the ability to modulate inflammation effectively. Translating laboratory research findings into clinical applications presents technical and regulatory obstacles, necessitating streamlined processes and interdisciplinary collaborations. Therefore, overcoming scalability, reproducibility, and cost-effectiveness challenges is essential for successful translation, alongside navigating regulatory requirements to ensure patient safety and efficacy.

Future research directions involve exploring novel drug delivery systems or bioactive molecules to enhance tissue regeneration and wound healing. In this context, targeted delivery systems such as nanoparticles would be highly advantageous, especially considering the influence of the protein corona on modulating their biological effects, inflammation responses and targetability as delivery systems.^200−202^ Moreover, personalized treatment strategies based on individual patient characteristics and wound types also represent a promising avenue for optimization. For instance, 3D-printed dressing materials offer personalized therapy for patients with wounds by delivering tailored dosage and physical properties, considering the patient’s clinical requirements and the type and characteristics of the wound.^203^ Another personalized therapy approach for diabetic wound healing using patients’ fibroblasts was demonstrated in a study by Santarella et al.; they illustrated that fibroblasts from diabetic ulcers could be stimulated to regenerate tissue when incorporated within an engineered ECM scaffold environment.^204^

The special attention given to the novel concept of personalized protein corona allows future experiments in this field, thereby facilitating the acceleration of clinical translation across various fields,^205^ potentially wound healing. The personalized protein corona concept originates from the understanding that the protein corona significantly influences various physicochemical and biological properties of nanoparticles. The protein corona composition is closely linked to the patient’s disease condition.^205^ Consequently, by identifying the protein corona profile of nanoparticles in individual patients, it becomes possible to engineer nanoparticles that offer personalized treatment/targeting. In wound healing, this approach may be utilized to optimize nanoparticle-based therapies, including targeted delivery of therapeutics, achieved by engineering nanoparticles with personalized protein corona.

Overall, while numerous preclinical studies are available related to inflammatory-modulating bioactive molecules and biomaterials for diabetic wound healing, further research efforts and collaborations between academia, industry, and healthcare providers are needed to overcome existing challenges and realize the full potential of these innovative therapies in improving outcomes for diabetic patients with chronic wounds.

## 7 Conclusions

The complex nature of diabetic wound healing necessitates a multifaceted approach targeting inflammatory dysregulation and leveraging innovative biomaterials and nutritional interventions. By understanding the intricate interplay of cytokines, immune cells, and molecular pathways involved in diabetic wound pathophysiology, researchers/clinicians can devise targeted strategies to accelerate healing and mitigate complications.

From interleukins like IL-22 and IL-1β to anti-inflammatory cytokines such as IL-10 and chemokines like CCL2, interventions targeting these molecular factors have shown promising results in diabetic wound healing. Polymeric-based, silk fibroin, stem cells, microRNAs, enzyme-based materials, and natural compounds like curcumin and quercetin, have demonstrated potential in modulating inflammation and promoting wound closure in diabetic patients.

While basic research has provided valuable insights into the mechanisms underlying diabetic wound healing, translating these findings into clinical practice remains challenging. Clinical trials investigating biomaterial-based therapies for diabetic wounds are relatively scarce, highlighting the need for more robust clinical evidence to support their efficacy and safety. Nevertheless, clinical studies conducted thus far have shown promising outcomes, indicating the potential of inflammation-modulating biomaterials in improving diabetic wound management. Further research and clinical trials are warranted to optimize these therapies and address the unmet needs in diabetic wound care.
